# New insights into the role of chrysanthemum calcineurin B–like interacting protein kinase CmCIPK23 in nitrate signaling in *Arabidopsis* roots

**DOI:** 10.1038/s41598-021-04758-8

**Published:** 2022-01-19

**Authors:** Bowen Liu, Hongmei Fan, Cuihui Sun, Mingyue Yuan, Xi Geng, Xiao Ding, Rui Ma, Na Yan, Xia Sun, Chengshu Zheng

**Affiliations:** 1 Department of Ornamental Horticulture, College of Horticulture Science and Engineering, Shandong Agricultural University, Tai’an, 271018 Shandong China; 2 Chrysanthemum Research Center of China, Japan and Korea in Shandong Province, Tai’an, 271018 Shandong China; 3 State Key Laboratory of Crop Biology, College of Life Sciences, Shandong Agricultural University, Tai’an, 271018 Shandong China

**Keywords:** Molecular biology, Plant sciences

## Abstract

Nitrate is an important source of nitrogen and also acts as a signaling molecule to trigger numerous physiological, growth, and developmental processes throughout the life of the plant. Many nitrate transporters, transcription factors, and protein kinases participate in the regulation of nitrate signaling. Here, we identified a gene encoding the chrysanthemum calcineurin B-like interacting protein kinase CmCIPK23, which participates in nitrate signaling pathways. In *Arabidopsis*, overexpression of *CmCIPK23* significantly decreased lateral root number and length and primary root length compared to the WT when grown on modified Murashige and Skoog medium with KNO_3_ as the sole nitrogen source (modified MS). The expression of nitrate-responsive genes differed significantly between *CmCIPK23*-overexpressing *Arabidopsis* (*CmCIPK23*-OE) and the WT after nitrate treatment. Nitrate content was significantly lower in *CmCIPK23*-OE roots, which may have resulted from reduced nitrate uptake at high external nitrate concentrations (≥ 1 mM). Nitrate reductase activity and the expression of nitrate reductase and glutamine synthase genes were lower in *CmCIPK23*-OE roots. We also found that CmCIPK23 interacted with the transcription factor CmTGA1, whose *Arabidopsis* homolog regulates the nitrate response. We inferred that *CmCIPK23* overexpression influences root development on modified MS medium, as well as root nitrate uptake and assimilation at high external nitrate supply. These findings offer new perspectives on the mechanisms by which the chrysanthemum CBL interacting protein kinase CmCIPK23 influences nitrate signaling.

## Introduction

Nitrogen (N) is one of the most important macronutrients for plant growth and development^1^; it is integral to the structure of key cellular macromolecules and participates in many plant physiological processes^2^. Nitrate (NO_3_^−^) is the primary source of N in well-aerated soils^3,4^. In *Arabidopsis thaliana*, NO_3_^−^ is acquired by members of four protein families [NITRATE TRANSPORTER 1/PEPTIDE TRANSPORTER (NRT1/PTR), NITRATE TRANSPORTER 2 (NRT2), CHLORIDE CHANNEL (CLC), and SLOW TYPE ANION CHANNEL (SLAC1/SLAH)]^5^, then transported throughout the entire plant^6^. A portion of the nitrate is transformed into nitrite and then into ammonium through the activities of nitrate reductase (NR) and nitrite reductase (NIR), respectively^7^. The ammonium resulting from nitrate reduction is combined with carbon to produce amino acids via glutamate dehydrogenase or the glutamine synthetase/glutamate synthase cycle (assimilation)^8^.

Nitrate also serves as a signal that triggers multiple biological responses and developmental processes, thereby regulating root and shoot development, germination, flowering, and its own transport and assimilation^9^. Different concentrations and distributions of nitrate have contrasting effects on lateral root (LR) development^10,11^. LR development makes an important contribution to overall root system development^3^. In general, LRs are more responsive to variations in nutrient availability than are primary roots (PRs)^12^. Indeed, LRs are one of the most important components of the root system: their growth can increase root biomass, enabling the plant to absorb more water and nutrients and providing better anchorage in the soil^13^. Nitrate signaling pathways can promote the initiation and development of lateral roots^12^ through a process that involves transporters (NRT1.1/NPF6.3, NRT2.1, NITRATE TRANSPORTER 2.2 (NRT2.2))^14,15^, transcription factors (MADS-box protein ANR, TGACG-BINDING FACTOR 1/4 (TGA1/4), NIN Like Protein 6/7 (NLP6/7)), several kinases (CALCINEURIN B-LIKE INTERACTING PROTEIN KINASE 8/23 (CIPK8/23), CALCIUM-DEPENDENT PROTEIN KINASE 10/30/32 (CPK10/30/32))^16^, and a number of plant hormones (i.e., auxin, ethylene, abscisic acid (ABA), and gibberellin)^17^.

CBL protein-interacting protein kinases (CIPKs) belong to a family of serine-threonine kinases that specifically target calcineurin B–like proteins (CBLs)^18^. Twenty-six *CIPK* genes and 34 *CIPK* genes have been identified in *Arabidopsis* and rice, respectively^19–21^. AtCIPK proteins consist of a conserved N-terminal catalytic kinase domain and a highly variable C-terminal regulatory domain. There is a typical activation loop in the N-terminal domain and an FISL or NAF motif (that interacts with AtCBLs), a PPI motif (that interacts with protein phosphatase 2Cs), and an unknown functional motif in the C-terminal domain^22,23^. Most CIPK proteins mediate responses to abiotic stresses such as high salinity, osmotic stress/drought, cold^23^, wounding, flooding^24^, low potassium (K^+^), and high pH^23^. CIPK proteins also participate in the ABA signaling pathway; in sensing and signaling related to ions^25^ such as potassium, nitrate, and magnesium (Mg^2+^)^26^; and in iron (Fe^2+^) homeostasis^27^. Notably, *CIPK8* positively regulates the nitrate-induced expression of primary nitrate response (PNR) genes, including nitrate transporter genes and genes involved in nitrate assimilation. The primary root of *Arabidopsis cipk8* mutants was longer when nitrate was either the sole nitrogen source or was applied together with ammonium (as NH_4_NO_3_)^28^. Nonetheless, the role of CIPK proteins in nitrate signaling remains to be fully characterized.

In *Arabidopsis*, the CIPK23 kinase acts as a central component in the acquisition and homeostasis of multiple ions, including potassium^29,30^, nitrate^31,32^, ammonium^33^, iron^27^, magnesium^26^, and the non-iron metals zinc (Zn^2+^) and manganese (Mn^2+^)^34^. A previous study demonstrated that the *Arabidopsis cipk23* mutant had greater PR and LR lengths and more LRs than the wild type (WT) after 10 days (d) of growth on Hoagland’s medium containing 2 mM KNO_3_^33^. CIPK23 was shown to phosphorylate CHL1 (NRT1.1/NPF6.3) to mediate nitrate sensing^31,35^ and regulate nitrate uptake under conditions of sufficient nitrate supply^36^. Nitrate availability and distribution strongly influence root development^37^. Ho et al.^31^ demonstrated that *AtCIPK23* plays a negative role in the PNR: following nitrate exposure, upregulation of the nitrate responsive gene *AtNRT2.1* was higher in *cipk23* mutants than in the wild type. However, little is known about the function of *CIPK23* in nitrate signaling.

Chrysanthemum (*Chrysanthemum morifolium*) is an important traditional flower^34^ and is primarily propagated by cutting. The root system of the chrysanthemum cutting comprises mainly adventitious roots (ARs) and LRs, and it relies on nitrogen fertilizer to grow^17^. Previous research on nitrate signaling has focused primarily on the PNR and root architecture^38–40^. In recent years, a few important nitrate regulatory genes that act in the PNR have been identified in *Arabidopsis*. However, little is known about precise nitrate signaling mechanisms in chrysanthemum. The TCP (TEOSINTE BRANCHED/CYCLOIDEA/PROLIFERATING CELL FACTOR) transcription factor gene *CmTCP20* and *CmANR1* have been reported to positively modulate LR development in chrysanthemum^17,41^. Gu et al.^42^ showed that CmNAR2 (NITRATE ASSIMILATION RELATED2) interacted with CmNRT2 to promote nitrate uptake. Chen et al.^43^ found that *CmCLCa* plays an important role in NO_3_^−^ storage in leaf vacuoles and is a candidate gene for the improvement of chrysanthemum N-starvation tolerance.

In this study, the CIPK gene *CmCIPK23* was isolated from chrysanthemum, and the root development of *CmCIPK23*-OE *Arabidopsis* plants was shown to be inhibited on modified MS medium. The expression of nitrate-responsive genes differed significantly between *CmCIPK23*-OE and WT plants after nitrate treatment. Nitrate uptake, content, and assimilation were lower in *CmCIPK23*-OE roots. We also found that CmCIPK23 interacted with the transcription factor CmTGA1, whose *Arabidopsis* homolog regulates the nitrate response. These findings offer new perspectives on the mechanism by which chrysanthemum *CmCIPK23* influences nitrate signaling.

## Results

### *CmCIPK23* responds to nitrate

**Figure 1:**
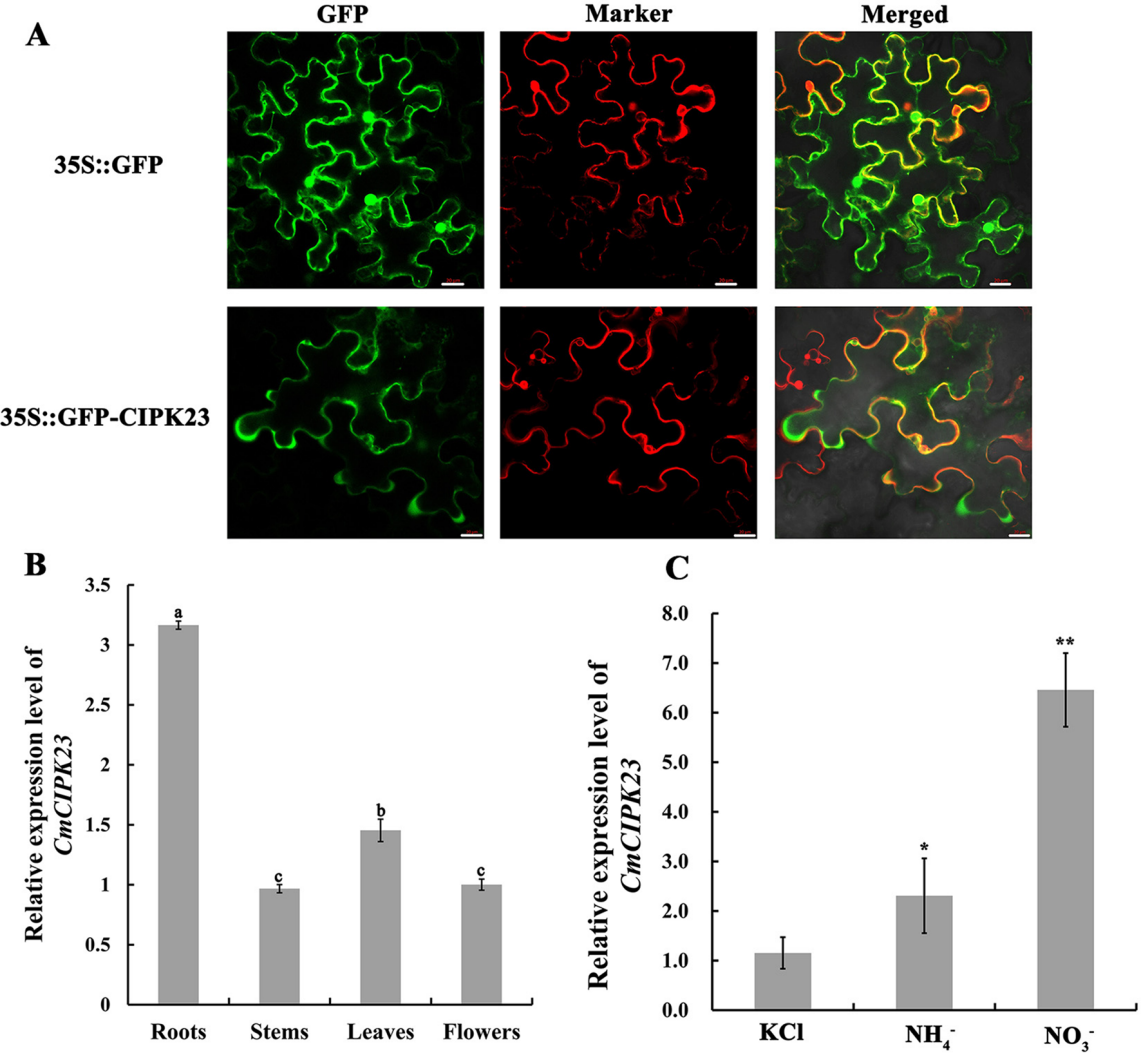
*CmCIPK23* responds to nitrate. (**A**) Subcellular localization of CmCIPK23. The pCaMV35S:*CmCIPK23-*GFP recombinant vector was transiently introduced into *N. benthamiana* leaves. The negative control was the pCaMV35S:GFP empty vector. The membrane marker CD-1007 (red) was obtained from the ABRC (*Arabidopsis* Biological Resource Center) and provided by Prof. Yong Wang (Shandong Agricultural University). Bars = 20 μm. (**B**) The relative expression of *CmCIPK23* in roots, stems, leaves, and flowers of adult chrysanthemum. The internal reference gene was *CmUbi*. Different letters indicate a significant difference [*P* < 0.05, least significant difference (LSD)]. (**C**) *CmCIPK23* transcript levels in roots treated with various nitrogen (N) sources after a 3-days period of N starvation. Cutting-propagated chrysanthemum plants were placed in 5 mM KCl, 5 mM NH_4_Cl, or 5 mM KNO_3_ for 3 h. The KCl-treated samples served as the controls. The internal reference gene was *CmUbi*. Each bar in (**B**) and (**C**) represents the mean ± SD of six replicates. n.s. *P* > 0.05. *P < 0.05. **P < 0.01 [Student’s *t*-test relative to KCl (**C**)].

The UN87566 fragment from chrysanthemum had previously been identified as homologous to *Arabidopsis* CIPK23 (AT1G30270)^44^. To ensure cloning sequence accuracy, we used nested 5′- and 3′-rapid-amplification of cDNA ends polymerase chain reaction (RACE PCR) to amplify this fragment. The full-length sequence was 1565 bp in length with a 1356-bp ORF (open reading frame), and NCBI SmartBLAST prediction indicated that it contained an STKc_SnRK3 serine/threonine kinase domain and a CIPK_C domain (Fig. S1A). Phylogenetic analysis of the chrysanthemum sequence and *Arabidopsis CIPK* family members showed that it was highly similar to *AtCIPK23* (AT1G30270) (Fig. S1B), and we therefore named it *CmCIPK23*. We transiently introduced a pCaMV35S:*CmCIPK23*-green fluorescent protein (GFP) fusion protein into *Nicotiana benthamiana* and used the empty vector as a negative control. Signal co-localization was observed by confocal laser scanning microscopy, and the GFP signal was not coincident with that of the cell membrane marker CD3-1007 (red) (Fig. 1A). These results demonstrated that CmCIPK23 was located in the cytosol (Fig. 1A).

Quantitative real time PCR (qPCR) expression profiling showed that *CmCIPK23* was expressed at a higher level in roots than in stems, leaves, and flowers of chrysanthemum (Fig. 1B). *AtCIPK23* was previously reported to be induced by nitrate and ammonium^31,33^, and we therefore treated chrysanthemum cutting-propagated seedlings with KNO_3_ or NH_4_Cl as a sole N source after N-starvation treatment. KCl-treated seedlings were used as controls. qPCR analysis showed that the expression of *CmCIPK23* in chrysanthemum roots was upregulated after exposure to NO_3_^−^ and ammonium (NH_4_^+^) (Fig. 1C). This result, together with the presence of conserved STKc_SnRK3 and CIPK_C domains, suggested that CmCIPK23 was a protein kinase that responds to NO_3_^−^ and NH_4_^+^.

### *CmCIPK23* inhibits nitrate-mediated root development when overexpressed in *Arabidopsis*

**Figure 2:**
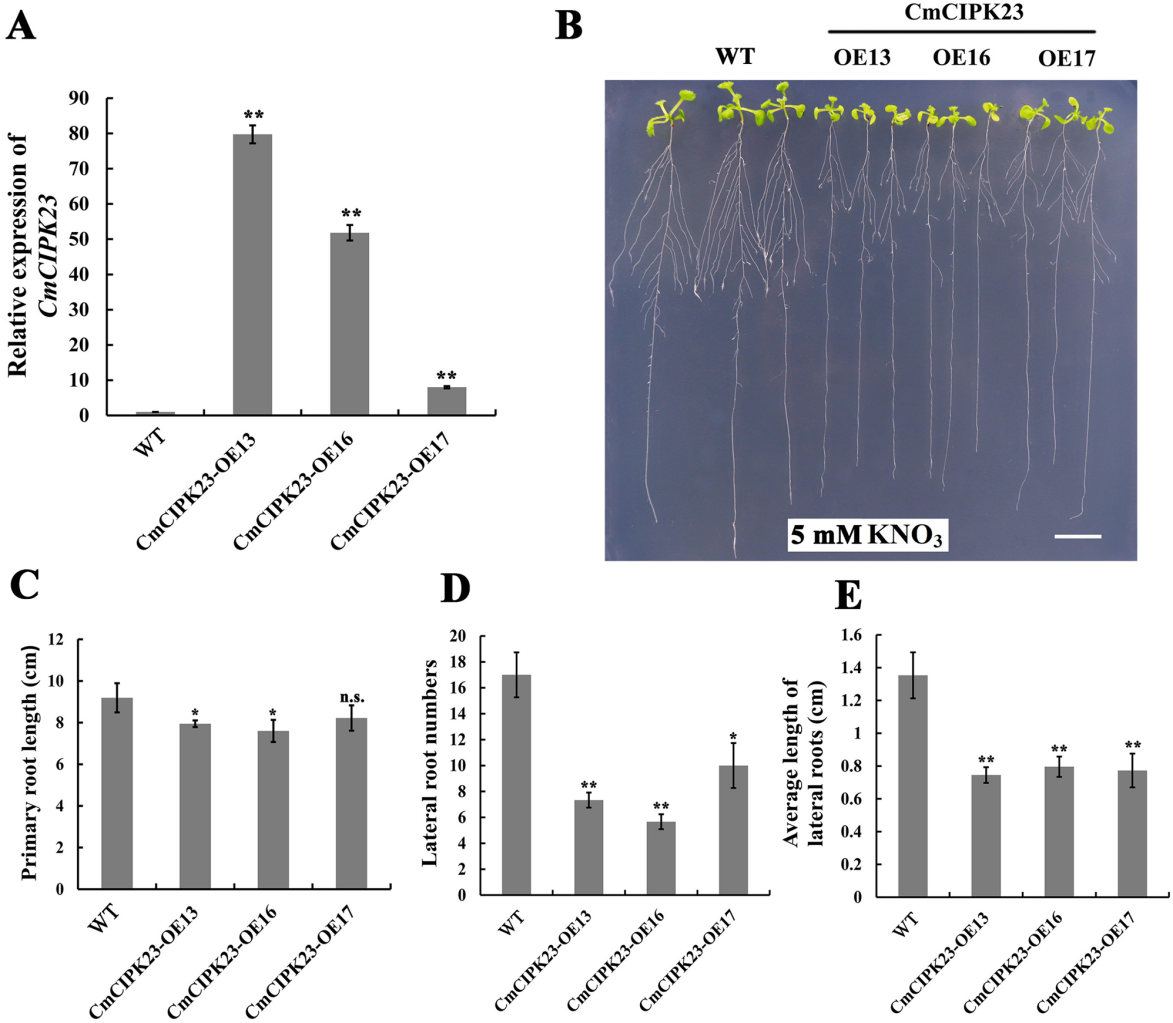
Heterologous overexpression of *CmCIPK23* in *Arabidopsis* inhibits root development. (**A**) Expression level of *CmCIPK23* in wild-type (WT) and three *CmCIPK23* transgenic *Arabidopsis* lines (*CmCIPK23*-OE lines). (**B**) Root developmental phenotypes of the WT and *CmCIPK23-*OE lines grown on modified MS medium with 5 mM KNO_3_ as the sole N source. Scale bar = 1 cm. (**C**) PR length, (**D**) number of LRs, and (**E**) average LR length of the WT and *CmCIPK23*-OE lines grown on modified MS medium that contained 5 mM KNO_3_. Each bar in (**C**)–(**E**) represents the mean ± SD of at least ten replicates. Three independent experiments were performed. n.s. *P* > 0.05. *P < 0.05. **P < 0.01 (Student’s *t*-test of individual OE lines versus WT).

To investigate the function of *CmCIPK23*, we constructed a 35S:*CmCIPK23*-GFP recombinant plasmid and introduced it into *Arabidopsis* by *Agrobacterium* GV3101-mediated transformation. After two generations of antibiotic selection, we obtained and identified three independent homozygous lines (*CmCIPK23*-OE13, -OE16, and -OE17) by qPCR (Fig. 2A), and we then assessed their root developmental phenotypes. WT and transgenic *Arabidopsis* seeds were plated onto modified MS medium containing 5 mM KNO_3_ as the sole N source and were grown vertically. After 10 days of growth, the transgenic seedlings had poorer root system development than the WT, with reduced PR lengths, lower average LR lengths, and fewer visible LRs (Fig. 2B–E). Specifically, the number and average length of LRs were 0.28–0.5-fold and 0.46–0.55-fold lower in the *CmCIPK23*-OE lines than in the WT (Fig. 2D,E). The overexpression lines also showed markedly reduced shoot development relative to the WT plants (Fig. 2B).

We next observed the phenotypes of WT and transgenic seedlings grown vertically for 10 days on modified MS medium with 0.25 mM KNO_3_ as the sole N source. As before, the number and average length of LRs were dramatically lower in *CmCIPK23*-OE lines than in the WT (Fig. S2). The number and average length of ARs were also lower in the *CmCIPK23*-OE lines (Fig. S3). These results suggest that *CmCIPK23* negatively regulates nitrate-mediated root development when overexpressed in *Arabidopsis* under conditions of both high and low nitrate supply.

### *CmCIPK23* affects the expression of nitrate-responsive genes in *Arabidopsis* roots

**Figure 3:**
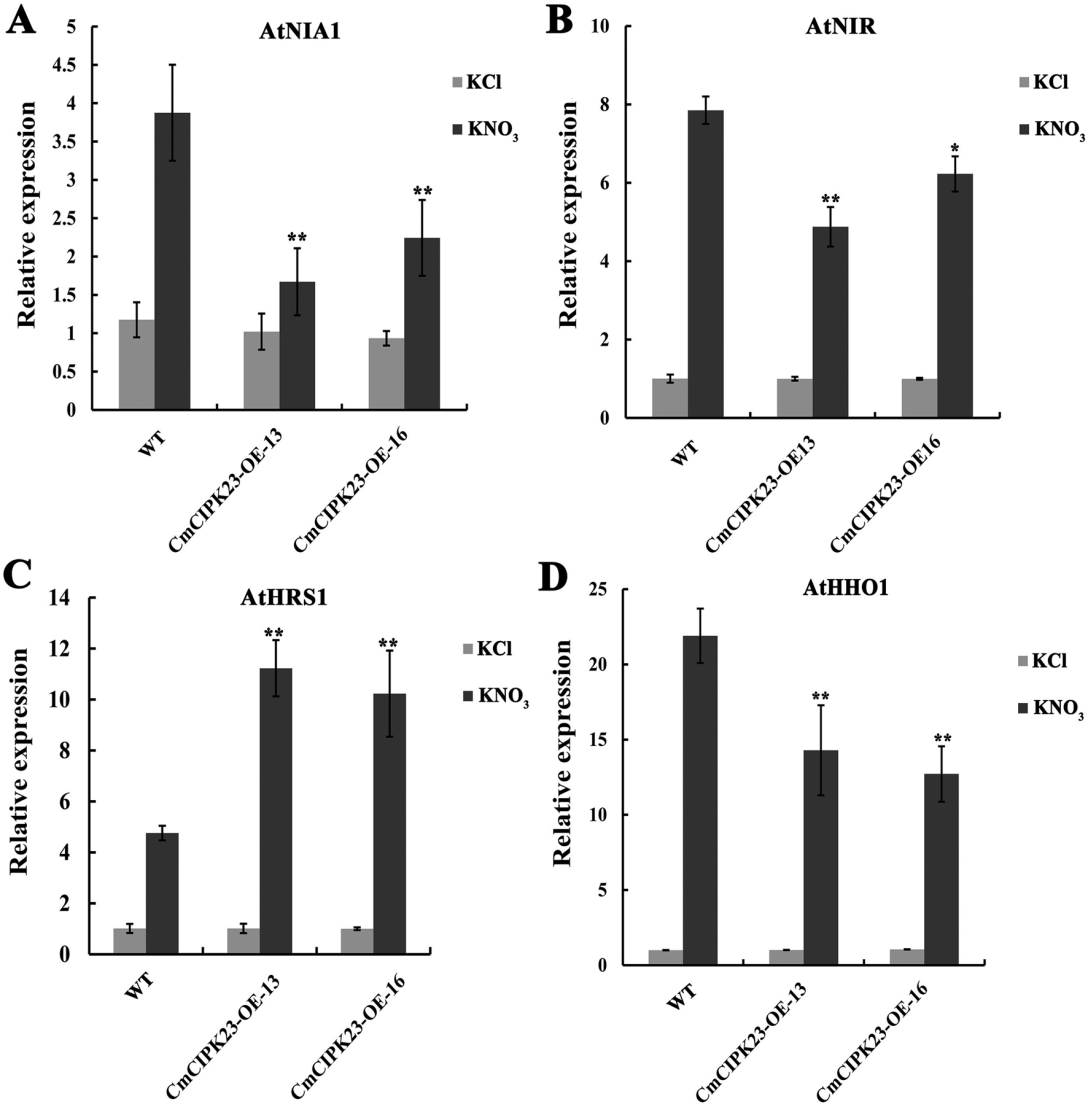
*CmCIPK23* affects the expression of nitrate-responsive genes in *Arabidopsis* roots. The expression of *AtNIA1* (**A**), *AtNIR* (**B**), *AtHRS1* (**C**), and *AtHHO1* (**D**) genes in the roots of the WT and *CmCIPK23*-OE lines. Seedlings were grown in solutions containing 2.5 mM ammonium succinate as the sole N source for 7 days, then treated with 5 mM KNO_3_ or 5 mM KCl (as a control) for 2 h. The relative expression levels of the four nitrate-responsive genes were measured using qPCR. Each column represents the mean ± SD of six replicates. n.s. *P* > 0.05. *P < 0.05. **P < 0.01 (Student’s *t*-test of individual OE lines vs. WT).

Nitrate response has been studied most extensively in roots, and *AtCIPK23* is a negative regulator of the PNR under low nitrate conditions^31,45^. The PNR refers to the rapid induction by nitrate of genes whose proteins are required for nitrate assimilation, nitrate transport, and energy and carbon metabolism^46–48^. These genes are regarded as primary nitrate-responsive genes. To determine whether *CmCIPK23* also participates in the PNR, we examined the nitrate-induced expression levels of the nitrate-responsive genes nitrate reductase 1 (*AtNIA1*) and nitrite reductase (*AtNIR*) and the nitrate signaling–related genes *AtHRS1* (*HYPERSENSITIVITY TO LOW PI-ELICITED PRIMARY ROOT SHORTENING 1*) and *AtHHO1 (HRS1 HOMOLOGUE 1)*. Recently, *AtHRS1* was shown to repress nitrogen starvation responses to optimize nitrogen acquisition and utilization under various levels of nitrogen availability and demand^49^. *AtHRS1* and *AtHHO1* were involved in repressing primary root growth when phosphate (P) was deficient and nitrate was present, suggesting that *HRS1*/*HHO1* act as integrators of P and nitrate signaling in the root tip^50^. As shown in Fig. 3A,B and D, the relative expression levels of *AtNIA1*, *AtNIR*, and *AtHHO1* were increased by nitrate treatment in the roots of all genotypes. However, their expression was 20–57% lower in the *CmCIPK23*-OE lines than in the WT under nitrate treatment (Fig. 3A,B,D). By contrast, the expression of *AtHRS1* showed a different trend (Fig. 3C). Its expression was also higher in response to nitrate in all genotypes, but it was 222–243% higher in the *CmCIPK23*-OE lines than in the WT under nitrate treatment (Fig. 3C). These results suggest that *CmCIPK23* affects the expression of nitrate-responsive genes and may have an important role in nitrate signaling.

### *CmCIPK23* influences nitrate uptake in *Arabidopsis* roots

**Figure 4:**
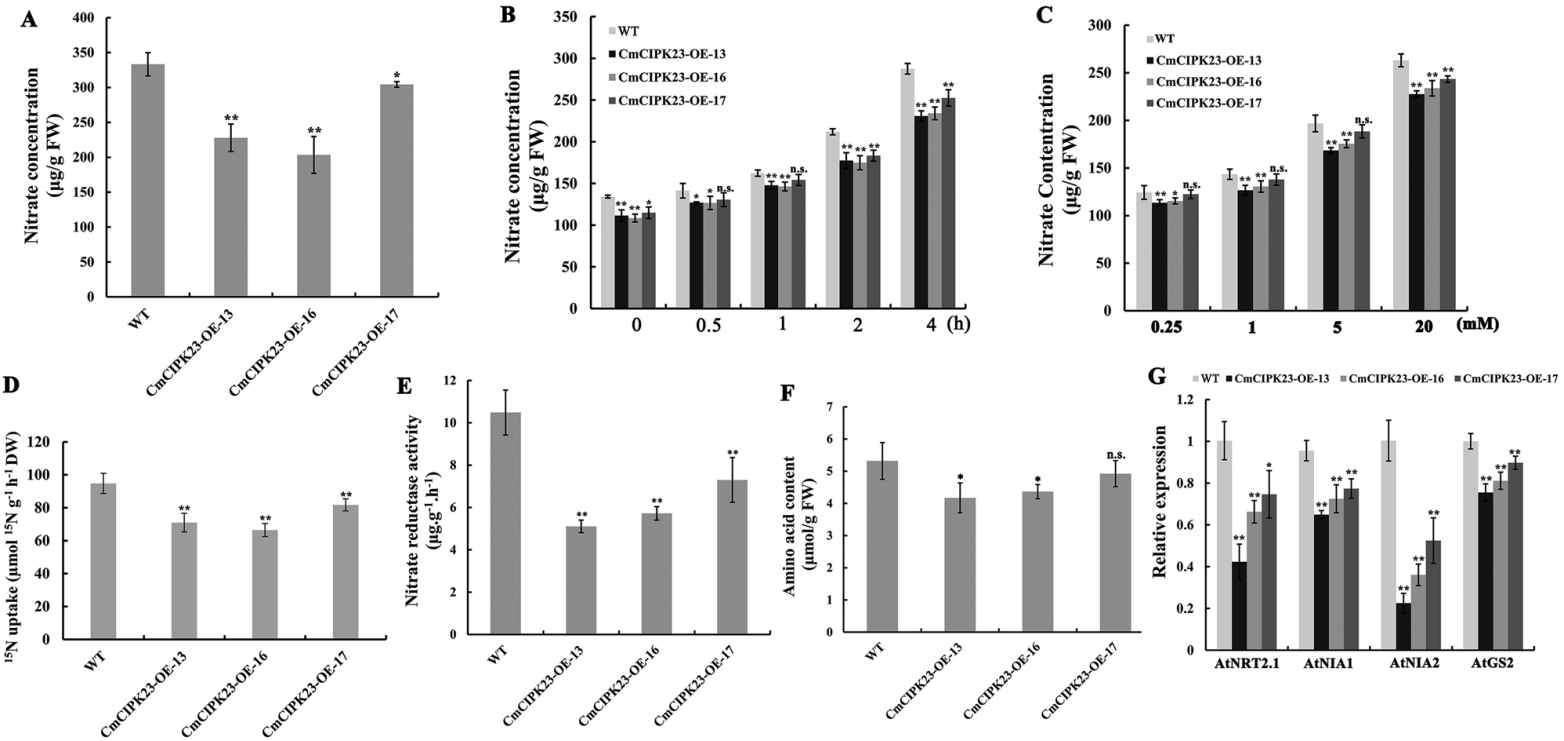
*CmCIPK23* regulates nitrate uptake and assimilation in *Arabidopsis* roots. Seedlings of WT and *CmCIPK23*-OE lines were grown in modified MS solution containing 5 mM KNO_3_ as the sole N source for 7 days. (**A**) The tissue nitrate concentrations of seedling roots. (**B**) The tissue nitrate concentrations of seedlings that were grown in nutrient solution with 2.5 mM ammonium succinate as the sole N source for 7 days, then moved to 5 mM KNO_3_ for the indicated time periods. (**C**) The tissue nitrate concentrations of seedlings that were grown in nutrient solution with 2.5 mM ammonium succinate as the sole N source for 7 days and then moved to various concentrations of KNO_3_ for 2 h. (**D**) ^15^NO_3_^−^ uptake in WT and *CmCIPK23*-OE lines. Seedlings were grown in modified MS solution for 7 days, then treated with 10 mM K^15^NO_3_ for 30 min. (**E**) Nitrate reductase activity in seedling roots. (**F**) Amino acid content in seedling roots. (**G**) The expression levels of nitrate transport and assimilation genes in seedling roots. Each bar represents the mean ± SD of six replicates. n.s. *P* > 0.05. *P < 0.05. **P < 0.01 (Student’s *t*-test of individual OE lines vs. WT).

Some nitrate regulators have been shown to affect nitrate accumulation in plant tissues^29,36,51,52^. To test the physiological effects of *CmCIPK23*, we measured the nitrate concentrations of seedling roots grown in the presence of 5 mM KNO_3_. Tissue nitrate concentrations were significantly lower in *CmCIPK23*-OE lines than in the WT (Fig. 4A). To test whether the reduced nitrate content was related to differences in nitrate absorption, we measured the nitrate content of seedlings grown in 2.5 mM ammonium succinate for 7 days and subsequently exposed to 5 mM KNO_3_ for different durations or to various concentrations of KNO_3_ for 2 h. Again, we found that root nitrate concentration was significantly lower in the *CmCIPK23*-OE lines (Fig. 4B,C). These results may arise from inhibited nitrate uptake in *CmCIPK23*-OE. We next performed a ^15^NO_3_^−^ uptake assay. WT and *CmCIPK23*-OE seedlings were grown in 5 mM KNO_3_ medium for 7 days and then treated with 10 mM K^15^NO_3_ for 30 min. ^15^NO_3_^−^ uptake was significantly lower in the *CmCIPK23*-OE lines compared with the WT (Fig. 4D). Taken together, these results suggest that *CmCIPK23* affects nitrate uptake at high external nitrate concentrations.

Previous research has shown that *AtNRT2.1* encodes a nitrate transporter involved in nitrate uptake^53–56^ and root development^57–59^. We next measured *AtNRT2.1* transcript levels after growing seedlings in modified MS solution containing 5 mM KNO_3_ for 7 days. qPCR analysis showed that the relative expression of *AtNRT2.1* was significantly lower in *CmCIPK23*-OE lines than in the WT (Fig. 4G), a result consistent with previous work on *CIPK23* in *Arabidopsis*^31^. Given the documented role of AtNRT2.1 in nitrate signaling, our data imply that reduced *AtNRT2.1* expression may contribute to lower nitrate uptake and impaired nitrate-mediated root development in *CmCIPK23*-OE lines.

Next, we measured NR activity and amino acid concentrations in seedling roots. Both were markedly lower in *CmCIPK23*-OE lines than in the WT (Fig. 4E,F). We also measured the expression of several important genes involved in nitrate assimilation (*AtNIA1, AtNIA2*, *AtNIR*, and *AtGS2*). The expression levels of *AtNIA1*, *AtNIA2*, and *AtGS2* (*GLUTAMINE SYNTHETASE 2*) were much lower in *CmCIPK23*-OE roots than in WT roots (Fig. 4G), although the expression of *AtNIR* did not differ significantly between the OE lines and the WT (data not shown). Reduced NR activity in the *CmCIPK23*-OE lines may result from the lower expression levels of *AtNIA1* and *AtNIA2*. Thus, *CmCIPK23* may affect nitrate assimilation in roots. All these findings demonstrate that *CmCIPK23* acts as an important regulator of nitrate uptake and assimilation in roots, controlling the nitrate content of roots by reducing nitrate absorption at high external nitrate concentrations.

### CmCIPK23 interacts with CmTGA1

**Figure 5:**
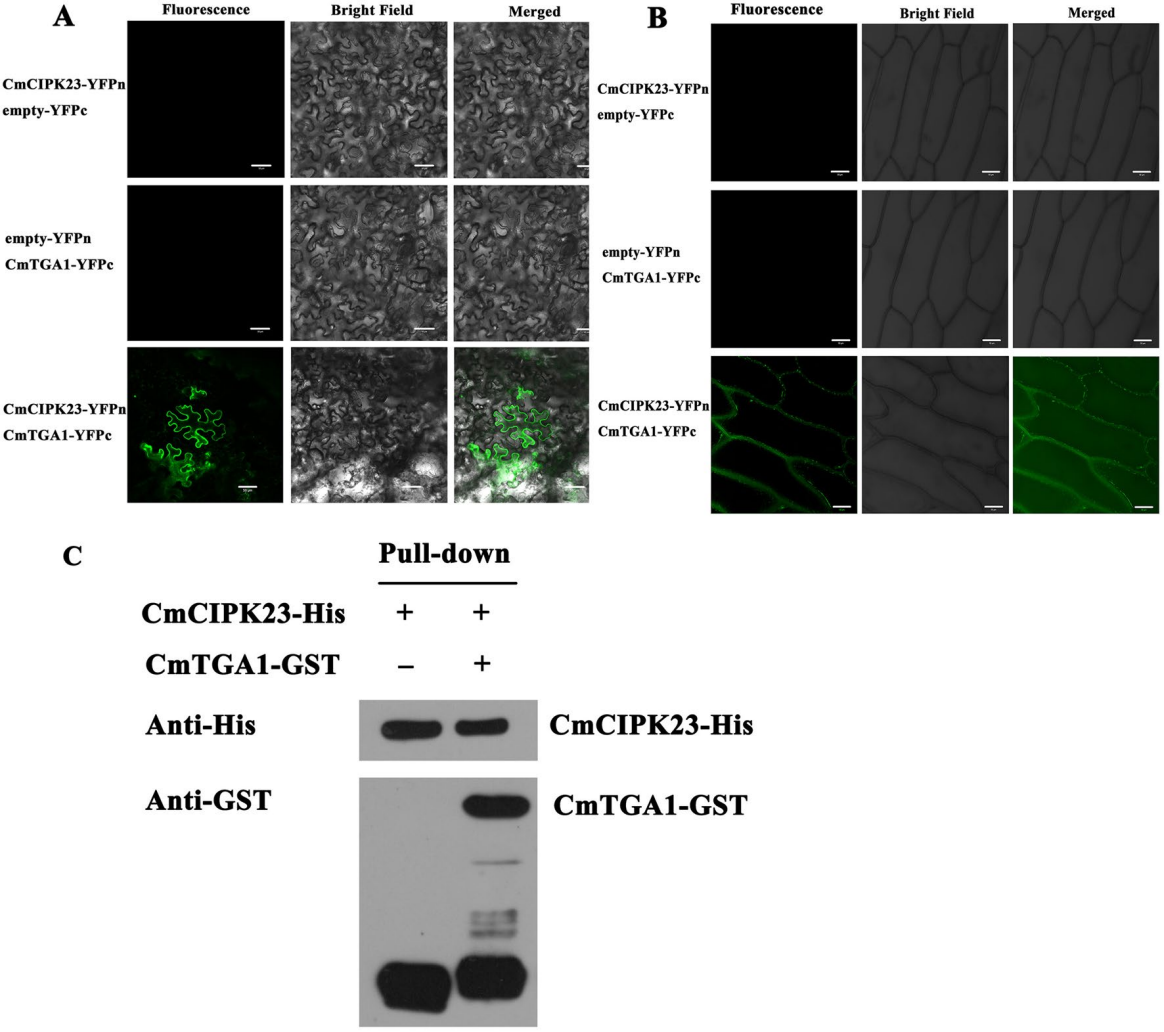
CmCIPK23 physically interacts with CmTGA1. CmCIPK23 interacted with CmTGA1 in BiFC assays. BiFC in *N. benthamiana* (**A**) and onion epidermal cells (**B**). The green signal is located in the plasma membrane. Scale bar = 50 μm. (**C**) His pull-down assay to verify the interaction between CmCIPK23 and CmTGA1 in vitro. The full-length western blot images are presented in Supplementary Fig. S4.

Previous research has identified TGA1 as an important transcription factor that regulates the nitrate response in *Arabidopsis* roots^60^. AtTGA1 has also been shown to interact directly with AtCIPK23^61^. We therefore investigated whether CmCIPK23 and CmTGA1 had a similar protein interaction using in vivo bimolecular fluorescence complementation (BiFC) assays (Fig. 5A,B) and in vitro His pull-down assays (Fig. 5C). In the BiFC assays, a direct interaction was observed between CmCIPK23 and CmTGA1 on the plasma membrane of *N. benthamiana* and onion epidermal cells when CmCIPK23-YFP^N^ was co-expressed with CmTGA1-YFP^C^ (Fig. 5A,B). In the pull-down assay, histidine (His)-tagged CmCIPK23 physically interacted with glutathione (GST)-tagged CmTGA1. GST-CmTGA1 was readily pulled down by cobalt affinity resin with His-CmCIPK23 and detected using an anti-GST antibody (Fig. 5C). These results confirm that CmCIPK23 interacts with CmTGA1.

## Discussion

NO_3_^−^ is the main N source for plants; it serves not only as a metabolic substrate for N assimilation but also as a signaling molecule that influences the expression of related genes to control plant growth and development^43,46,62^. The kinase CIPK23 controls the regulation of diverse root nutrient transporters^63^. Previous studies have shown that *Arabidopsis CIPK23* is a central component of nitrate signaling pathways^31,62^ and inhibits ammonium transport^33,64^. Here, *CmCIPK23* expression was rapidly upregulated by exogenous NO_3_^−^ and, to a lesser extent, by NH_4_^+^, consistent with previous results (Fig. 1C). To explore the role of chrysanthemum *CmCIPK23* in nitrate signaling, we generated three *CmCIPK23* over-expressing *Arabidopsis* lines (Fig. 2A). The earliest stages of the PNR in higher plants include the induction of genes involved in nitrate assimilation, nitrate transport, and related processes^65^. The PNR is accompanied by changes in nitrate transport activity and remobilization and by modulation of root growth^66^. Here, we found that the nitrate-induced transcript levels of the PNR genes *AtNIA1, AtNIR, AtHHO1*, and *AtHRS1* differed significantly between the *CmCIPK23*-OE lines and the WT (Fig. 3). These results demonstrate that *CmCIPK23* responds to nitrate and affects the primary nitrate response.

In *Arabidopsis*, the *cipk23* mutant exhibited more extensive root development than the WT after 10 days of growth on medium containing 2 mM KNO_3_^33^. Previous research demonstrated that *CIPK23* affected root development under conditions of extreme nutrient stress. For example, root length was shorter in the *cipk23* mutant than in the WT under low K^+^ (micromolar range)^67^ and under high ammonium levels^33^. *CIPK23* may also enhance stress tolerance to maintain normal root development. Here, we found that overexpression of *CmCIPK23* inhibited root development when seedlings were grown with KNO_3_ as the sole N source (Fig. 2, S2 and S3), and these results suggest that *CmCIPK23* participates in nitrate-mediated root development in *Arabidopsis*.

We also observed reduced shoot development in the *CmCIPK23*-OE lines relative to the WT (Fig. 2B). In addition to reduced nitrate uptake and assimilation, decreased translocation of nitrate to the shoot may also underlie this difference in shoot growth. *NRT1.1* is phosphorylated by *CIPK23* in *Arabidopsis*^31^, but its documented role in NO_3_^−^ translocation from roots to shoots may involve its nonphosphorylated form^68,69^. It is therefore possible that *CIPK23* overexpression increased the phosphorylation levels of NRT1.1, reducing its activity in nitrate translocation. In the future, it will be interesting to explore whether *CmCIPK23* regulates the translocation of nitrate between plant tissues.

Nitrate content was much lower in *CmCIPK23*-OE roots than in the WT (Fig. 4A), similar to findings in an *Arabidopsis AtCIPK23* overexpression line^36^. Nitrate uptake was also significantly lower in *CmCIPK23*-OE plants (Fig. 4B–D) at high external nitrate concentrations. Phosphorylation by *AtCIPK23* has been shown to activate the nitrate-sensing function of NRT1.1 and switch it from a low-affinity to a high-affinity nitrate transporter^35,70^. Overexpression of *CIPK23* may therefore promote *NRT1.1* phosphorylation, enhancing its high-affinity nitrate transport activity even under sufficient nitrate supply and thereby reducing nitrate absorption^36^. In addition, the overexpression of *CmCIPK23* at high external nitrate concentrations also inhibited the relative expression of *AtNRT2.1* (Fig. 4G), which has a key role in the regulation of root high-affinity NO_3_^−^ uptake^55,56^. In recent years, numerous reports have focused on the function of NRT2.1 in nitrate uptake. The interaction of NRT2.1 with the NAR2 protein^71^ plays a major role in root nitrate uptake of many plants and has been studied extensively in wheat and the primary root of maize^54,55,72–75^. NRT2.1 is phosphorylated at multiple sites^76–78^, and it will be important to determine whether CIPK23 influences nitrate uptake through phosphorylation of NRT2.1. *NRT2.1* has also been shown to have a positive effect on root growth^57,58^, but recent research suggests that the positive influence of NRT2.1 on lateral root formation occurs only on medium with NO_3_^−^ as the sole N source^59^. A possible explanation for this result is that ammonium causes lateral root outgrowth by acidifying the root apoplast to increase the pH-dependent import of protonated auxin^79^. This may also counteract the strong inhibition of *NRT2.1* expression by ammonium^14,80^, and negative effects of reduced NRT2.1 on lateral root development would therefore be counteracted by pH-driven auxin transport^59^. In the present study, we observed that impaired root development in *CmCIPK23*-OE lines occurred only on medium with NO_3_^−^ as the sole N source (Fig. 2 and S2). Also, *AtNRT2.1* expression was downregulated in *CmCIPK23*-OE lines only when grown on medium with NO_3_^−^ as the sole N source (Fig. 4G). Our results confirm that *CmCIPK23* overexpression affects nitrate-mediated root development and nitrate uptake, but the presence of additional N forms may create a more complex scenario.

The assimilatory nitrate reduction pathway is a very significant physiological process, as it is one of the main routes by which inorganic nitrogen is assimilated into carbon skeletons to regulate the growth and development of higher plants^81^. Here, NR activity and amino acid content were also lower in *CmCIPK23*-OE roots relative to the WT (Fig. 4E,F). We found that the expression levels of *AtNIA1*, *AtNIA2*, and *AtGS2* were downregulated in *CmCIPK23*-OE roots compared with the WT (Fig. 4G), and this probably contributed to observed reductions in nitrate assimilation. Based on previous results, we infer that *CmCIPK23* affects nitrate assimilation in roots.

TGA1, a basic region/leucine zipper motif (bZIP) family transcription factor, has been identified as an important regulator of the nitrate response in *Arabidopsis* roots^60^. The *tga1/tga4* double mutant shows impaired responses to NO_3_^−^ treatment, including modulation of primary root length and lateral root density^60^. TGA1 has been shown to bind to the *NRT2.1* and *NRT2.2* promoters in chromatin immunoprecipitation (ChIP) assays, thereby controlling the expression of these two high-affinity nitrate transporter genes. In this study, we found that CmCIPK23 interacted with CmTGA1 (Fig. 5), and the expression of *AtNRT2.1* was significantly lower in *CmCIPK23*-OE lines than in the WT (Fig. 4G). These results indicate that CmTGA1 is a putative partner of CmCIPK23 in the regulation of nitrate responses. In addition, NO_3_^−^-induced expression of *TGA1* is also dependent on phospholipase C (PLC)-calcium signaling downstream of *AtNRT1.1*^82^. Considering the interaction of CIPK23 and NRT1.1^31,70^, the TGA1–CIPK23 interaction demonstrated here suggests that NO_3_^−^-mediated activation of CmTGA1 may occur in an AtNRT1.1-dependent manner^61^.

*Chrysanthemum morifolium* is a commercially important ornamental species that requires sufficient nitrogen fertilizer to promote vegetative growth^17,83^. *Chrysanthemum morifolium* culture demands a strong root system with highly efficient N uptake. Nonetheless, the response of nitrate regulatory genes to nitrate signaling in chrysanthemum has not been fully investigated. Here, we found that *CmCIPK23* expression responds to nitrate and that the CmCIPK23 protein is localized in the cytosol. *CmCIPK23* overexpression in *Arabidopsis* roots reduced nitrate uptake, and this reduced uptake contributed to lower tissue nitrate concentrations despite high external nitrate supply. CmCIPK23 was also shown to interact physically with the transcription factor CmTGA1, whose *Arabidopsis* homolog regulates the nitrate response. These results strongly suggest that *CmCIPK23* is an important regulator of the nitrate response. These new insights into the functions of *CmCIPK23* in nitrate signaling are just the beginning of unraveling the nitrate signaling network in chrysanthemum, but they have implications for the breeding of strongly rooted, nitrate-efficient chrysanthemum varieties. It will be interesting to investigate the functions and mechanisms by which *CIPK23* and its putative partner *TGA1* participate in nitrate signaling.

## Materials and methods

### Growth and treatment of plant materials

*Chrysanthemum morifolium* ‘Jinba’ cuttings from Prof. Junping Gao (China Agricultural University, China), approximately 10 cm in height, were cultivated in a 1:1 mixture of perlite and vermiculite under a 16-h photoperiod (∼ 100 μmol/m^2^/s) at 22 ± 1 °C^17^. After 20 days, we selected rooted cuttings with similar heights and diameters and exposed them to sterile water for 3 days as an N starvation treatment. The N-starved seedlings were then divided into three groups of 15 seedlings each and exposed to improved Hoagland’s nutrient solution that contained either 5 mM KNO_3_, 5 mM NH_4_Cl, or 5 mM KCl for 3 h. The improved Hoagland’s nutrient solution has been described in a previous paper^61^. The roots of the treated seedlings were collected and stored in an ultra-low temperature freezer (− 80 °C) for use in qPCR.

*Arabidopsis* experiments were performed with the *Arabidopsis thaliana* ecotype ‘Columbia’ from Prof. Yujin Hao (Shandong Agricultural University, China). Seeds were sterilized by soaking in rubbing alcohol (75% alcohol by volume) for 5 min and in 2.5% sodium hypochlorite for 10 min. The seeds were then rinsed and vernalized at 4 °C for 3 days. Seeds were plated on modified MS solid medium (Coolaber, China) containing 0.5% (w/v) sucrose, 1% (w/v) agar, and 0.25 mM or 5 mM KNO_3_ as the sole N source. Approximately ten seeds were placed on each plate, and there were ten plates per treatment. Seeds were germinated at 22 ± 1 °C under a 16-h light/8-h dark photoperiod. After 10 days of vertical cultivation, we observed the root developmental phenotypes of the WT and *CmCIPK23*-OE plants (see below).

Seeds of WT and *CmCIPK23*-OE13/16/17 lines were germinated on modified MS medium containing 5 mM KNO_3_ as the sole N source. Hypocotyls were removed from 5-days-old seedlings, the seedlings were grown vertically in the dark for 7 days, and their adventitious root development phenotype was then observed.

For gene expression profiling, we cultivated seedlings in six-well plates with 35–40 seeds per well; each plate contained a line. *CmCIPK23*-OE seedlings were grown in ammonium succinate solution that contained modified MS, 0.5% (w/v) sucrose, and 2.5 mM ammonium succinate as the sole N source for 7 days. They were then treated with 5 mM KNO_3_ or 5 mM KCl for 2 h, and the transcript levels of nitrate regulatory genes were measured by qPCR (see below). Seedlings were also harvested from plants grown on modified MS medium with 5 mM KNO_3_ for 7 days and used to measure aspects of nitrogen metabolism and the expression levels of nitrate-related genes.

### RNA extraction and cloning of *CmCIPK23*

We extracted total RNA from roots of chrysanthemum ‘Jinba’ using the TRIzon reagent (CWBIO, China). The purified RNA was converted to RACE-ready cDNA using the SMARTer RACE 5′/3′ Kit (Clontech, USA). The full length of *CmCIPK23* cDNA was amplified with the GSP3′ and GSP5′-1/2 RACE primers and Super-Fidelity DNA Polymerase (Vazyme, China). The targeted PCR product was cloned into a *pEASY* cloning vector (TransGen, China) for sequencing analysis. All primer sequences are shown in Table S1.

For phylogenetic analysis, we used DNAMAN (https://www.lynnon.com) to construct a multiple alignment of CmCIPK23 and CIPK23/CIPK23-like protein sequences from other species. We used the protein sequences of CmCIPK23 and CIPK family members from *Arabidopsis* to construct a neighbor-joining phylogenetic tree in MEGA7^84^.

### Subcellular localization and BiFC assays

For subcellular localization of CmCIPK23, the coding region of *CmCIPK23* was amplified using CIPK23-1258-F and CIPK23-1258-R primers (Table S1) and then joined to the 1258-35S vector. The 35S:*CmCIPK23*-GFP recombinant plasmid was transiently transformed into *Nicotiana benthamiana* epidermal cells, and GFP fluorescence was observed using a confocal microscope (Zeiss LSM880 Airyscan, Jena, Germany).

For the BiFC assay, the ORFs of *CmCIPK23* and *CmTGA1* were cloned into the pSPYNE-35S (YFP N-terminal portion) and pSPYCE-35S (YFP C-terminal portion) vectors, respectively. These constructs were co-transfected into *N. benthamiana* and onion epidermal cells, and the empty vectors YFP N and YFP C were used as negative controls. The fluorescence signal was observed using a confocal microscope (Zeiss LSM880 Airyscan, Jena, Germany).

### Generation of transgenic *Arabidopsis* lines overexpressing *CmCIPK23*

Chrysanthemum currently exhibits very low transformation efficiency, and we therefore made preliminary investigations of *CmCIPK23* function in *Arabidopsis*, recognizing that its behavior in *Arabidopsis* may not precisely mimic its behavior in chrysanthemum. We transformed the 35S:*CmCIPK23* recombinant plasmid into the *Arabidopsis* ecotype Columbia by the floral dip method using *Agrobacterium* strain GV3101^85^. Seedlings carrying the transgene in the homozygous state were identified after culturing the plants on MS medium containing 30 mg L^−1^ hygromycin for two generations. qPCR was used to measure the transcript level of *CmCIPK23*, and homozygous transgenic seeds were used for subsequent experiments. qPCR primer sequences are provided in Table S1.

### His pull-down assay

The ORFs of *CmCIPK23* and *CmTGA1* were cloned into the pET-28a and pGEX-6P-1 vectors, which contained a histidine (His) tag or glutathione S-transferase (GST) sequence, respectively. The recombinant plasmids were separately transformed into *Escherichia coli* strain Rosetta (DE3) or BL21 (DE3). The immobilization of the bait protein (His-CmCIPK23), the capture of the prey protein (GST-CmTGA1), and the bait-prey elution were performed using a His Pull-Down Kit (Thermo Fisher, USA). The prepared eluent was separated using 12% SDS–PAGE, transferred to polyvinylidene fluoride (PVDF) membranes (Millipore, Billerica, MA, USA), and probed with anti-GST antibody (Sigma-Aldrich, Merck KGaA, Darmstadt, Germany).

### Nitrogen measurements and nitrate reductase assay

Plant nitrate content was measured using the salicylic acid method as described previously^29^. Nitrate reductase activity was measured by the sulfanilamide colorimetric method^86^. In brief, samples (< 0.1 g) were milled into a powder and combined with 1 mL extraction buffer (pH 7.5) (25 mL 0.1 M phosphate buffer, 0.061 g cysteine, 0.186 g EDTA-2Na, 75 mL water), then centrifuged at 4 °C and 4000 g for 5 min. The supernatant (100 µL) was combined with 375 µL KNO_3_/0.1 M phosphate buffer (1/100, w/v) and 125 µL NADH/0.1 M phosphate buffer (0.002/1, w/v) in a glass test tube and incubated at 25 °C for 30 min in the dark. Two hundred fifty microliters of sulfanilic acid (1/100, w/v) and 250 µL α-naphthylamine (0.2/100, w/v) were added to the tube. The absorbance was measured at 540 nm and compared with that of a blank reference, and the nitrate reductase activity in the sample was calculated (UV-5200 spectrophotometer, METASH).

Amino acid content in plant tissue was measured by ninhydrin colorimetric analysis^87^. Samples (< 0.1 g) were milled into a powder and combined with 1 mL 10% acetic acid. After centrifuging at 13,000 rpm for 5 min, the supernatant was combined with 9 mL 0.2 M sodium acetate buffer (pH 5.4) and boiled for 15 min for amino acid extraction. One milliliter of extracted amino acids, 1 mL 0.2 M acetic acid buffer (pH 5.4), 3 mL ninhydrin solution, and 0.1 mL 0.3% ascorbic acid were combined in a tube, boiled at 100 °C for 15 min, and then combined with 4.9 mL 0.2 M acetic acid buffer (pH 5.4). The absorbance was measured at 570 nm and compared with that of a blank reference, and the amino acid content in the sample was calculated.

### Analysis of nitrate uptake using ^15^NO_3_^−^

^15^NO_3_^−^ uptake was measured as described by Wang and Tsay^52,88^. We cultivated seedlings in six-well plates with 35–40 seeds per well; each plate contained a line. *CmCIPK23*-OE seedlings were grown in 5 mM KNO_3_ solution that contained modified MS, 0.5% (w/v) sucrose, and 5 mM KNO_3_ as the sole N source for 7 days. They were then treated with 10 mM K^15^NO_3_ (98% atom ^15^ N, Sigma, USA) for 30 min and washed in 0.1 mM CaSO_4_ for 1 min. The seedlings were dried at 80 °C for 2 days. ^15^N content was analyzed on a stable isotope ratio mass spectrometer (Elementar, ISOprime 100, UK).

### Real-time PCR analysis

Total RNA was purified as described above, and first-strand cDNA was synthesized using the HiFiScript gDNA Removal cDNA Synthesis Kit (CWBio, China). Real time qPCR assays were performed using ChamQ Universal SYBR qPCR Master Mix (Vazyme, China) on the LightCycler 480 II instrument (Roche, USA). Relative gene expression was calculated by the 2^−∆∆Ct^ method using *CmUbi* as the internal reference gene. The thermal cycler program consisted of 95 °C for 8 min and 40 cycles of 95 °C for 15 s and 60 °C for 1 min. The primers used in the qPCR reactions are provided in Table S1.

### Statistical analyses

All data are presented as mean ± standard deviation (SD), and unless otherwise specified, differences between treatments or genotypes were assessed using Student’s *t*-test (*P* < 0.05) performed with SPSS 22.0 software (IBM Corp., Armonk, NY, USA).

### Permission statement for plant materials

All required approvals for the collection of plant or seed specimens were obtained for this study, which complied with relevant institutional, national, and international guidelines and legislation.

## Supplementary Information

Supplementary Information.

## Data Availability

The data produced in this research may be obtained from the corresponding authors upon reasonable request.
