# Supplementary material for: New insights into the role of chrysanthemum calcineurin B–like interacting protein kinase CmCIPK23 in nitrate signaling in Arabidopsis roots
Source: Sci Rep. 2022 Jan 19;12:1018. doi: 10.1038/s41598-021-04758-8 (PMC8770472; doi:10.1038/s41598-021-04758-8)
Supplement: Supplementary file 1 — Supplementary Information. [file 41598_2021_4758_MOESM1_ESM.pdf]

New insights into the role of chrysanthemum calcineurin B–like interacting protein kinase CmCIPK23 in nitrate signaling in *Arabidopsis* roots

Supplemental Information

Bowen Liu[1,2], Hongmei Fan[3], Cuihui Sun[1,2], Mingyue Yuan[1,2], Xi Geng[1,2], Xiao Ding[1,2], Rui Ma[1,2], Na Yan[1,2], Xia Sun[1,2] *& Chengshu Zheng[1,2] *

[1]*Department of Ornamental Horticulture, College of Horticulture Science and Engineering, Shandong Agricultural University, Tai'an, Shandong 271018, China*

[2]*Chrysanthemum Research Center of China, Japan and Korea in Shandong Province, Tai'an, Shandong 271018, China*

[3]*State Key Laboratory of Crop Biology, College of Life Sciences, Shandong Agricultural University, Tai'an, Shandong 271018, China*

*To whom correspondence should be addressed. E-mail: zcs@sdau.edu.cn; sunxia65@sina.com

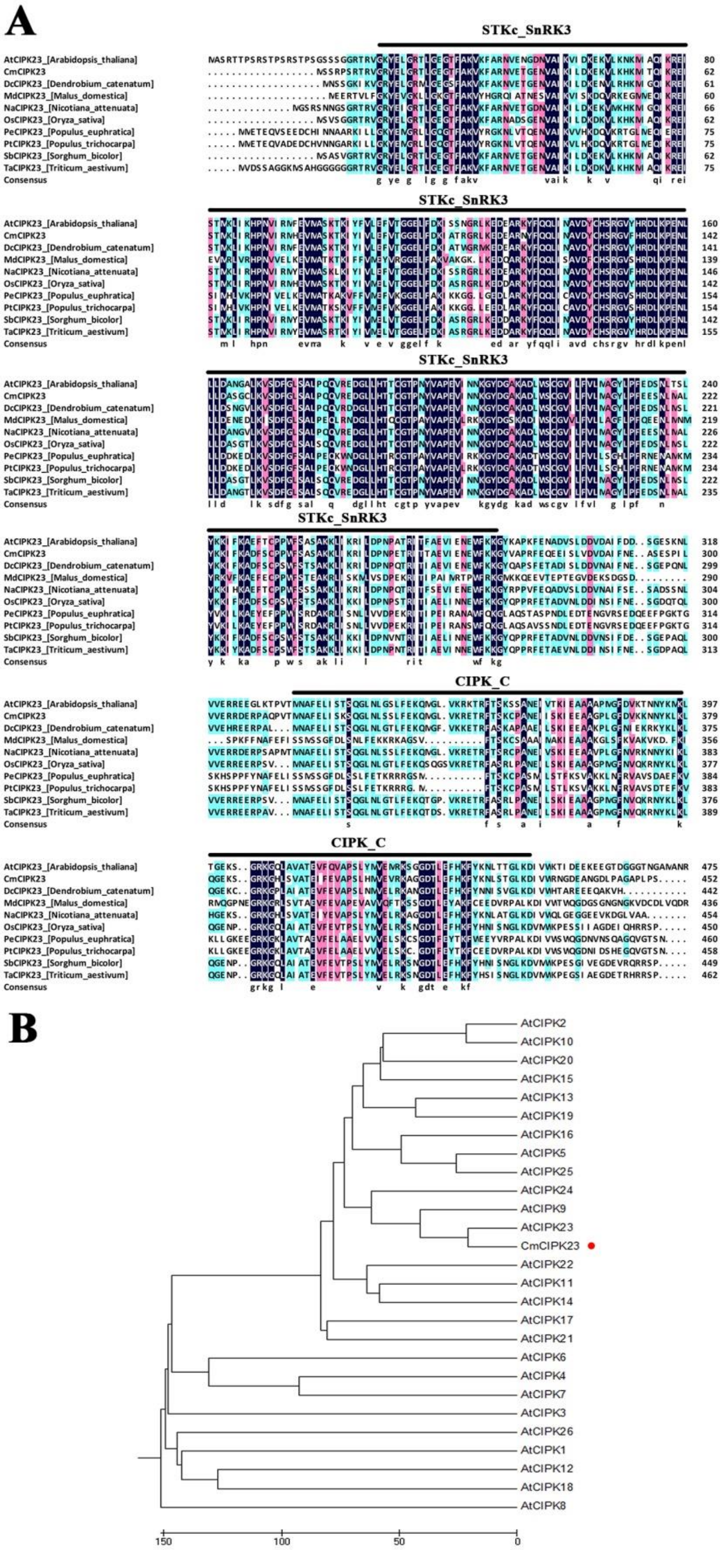

Fig. S1. Analysis of the deduced amino acid sequence of *CmCIPK23*. (A) Multiple alignment of STKc_SnRK3 and CIPK_C domains of CmCIPK23 and CIPK23/CIPK23-like genes of other species. (B) Phylogenetic tree of the protein sequences of CmCIPK23 and members of the CIPK family from *Arabidopsis*. The tree was constructed in MEGA7 by the neighbor-joining method. The bar indicates the unit length of divergence between

sequences.

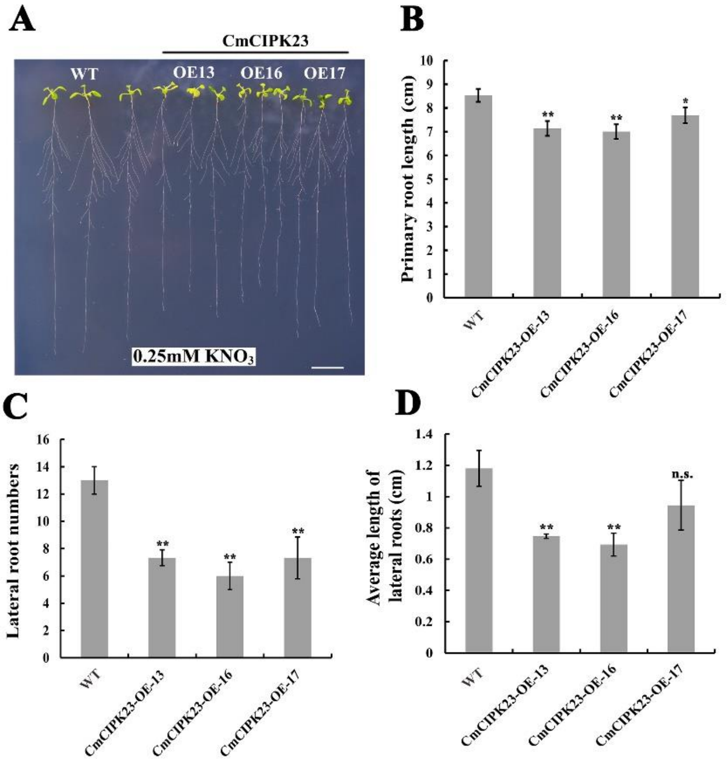

Fig. S2. Heterologous overexpression of *CmCIPK23* in *Arabidopsis* inhibits root development. (A) Root systems of 10-day-old WT and *CmCIPK23*-OE lines grown on modified MS medium with 0.25 mM KNO_3 as the sole N source. Scale bar = 1 cm. (B) PR length, (C) number of LRs, and (D) average LR length of the WT and *CmCIPK23*-OE lines on modified MS medium with 0.25 mM KNO_3. Each bar in (B)–(D) represents the mean ± SD of at least ten replicates. Three independent experiments were performed. n.s. $P > 0.05$. *$P < 0.05$. **$P < 0.01$ (Student's *t*-test of individual OE lines versus WT).

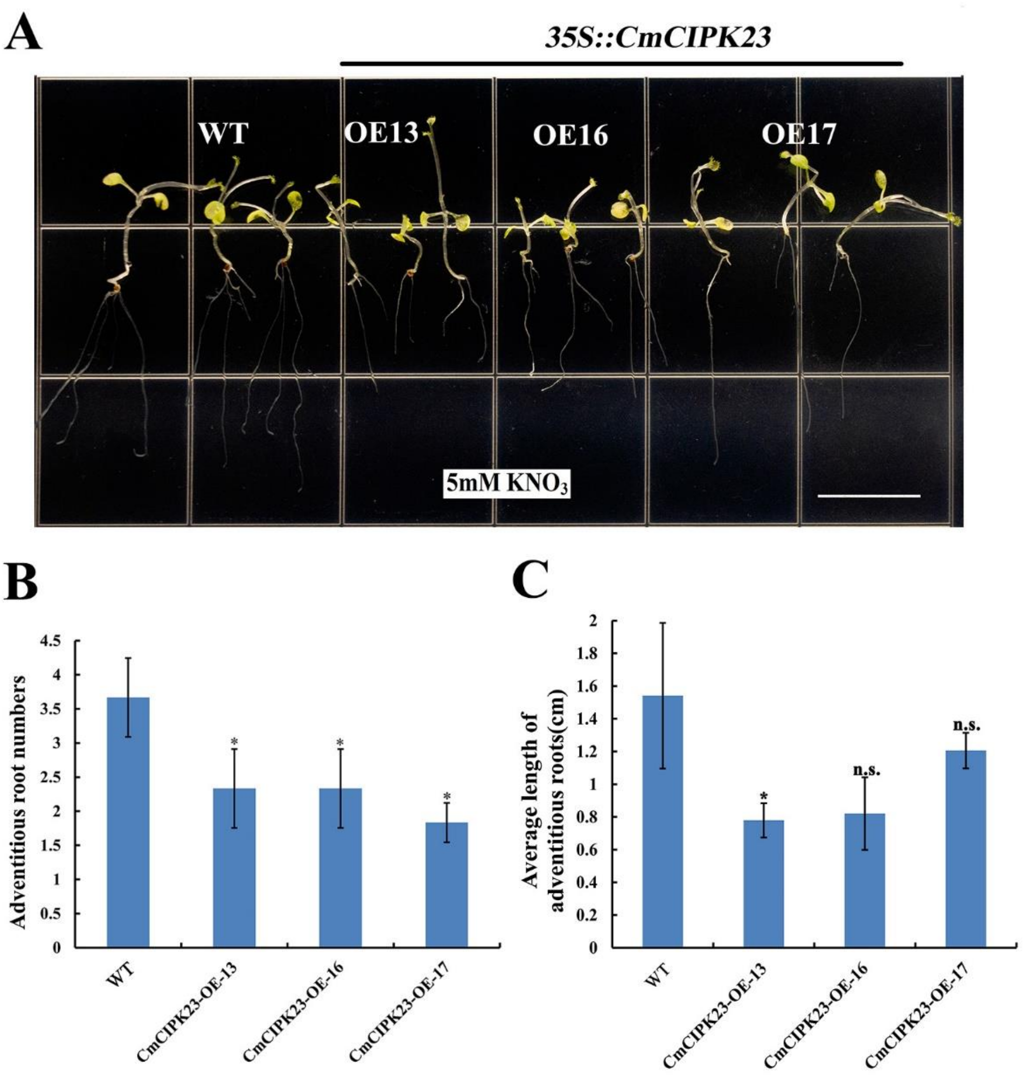

Fig. S3. Adventitious rooting (AR) on hypocotyls of WT and *CmCIPK23*-OE lines under dark conditions. (A) AR phenotypes of WT and *CmCIPK23*-OE lines grown for 7 d on modified MS medium with 5 mM KNO_3 as the sole N source under dark conditions. Scale bar = 1 cm. (B) The number and (C) average length of ARs in the WT and *CmCIPK23*-OE lines. Each bar in (B) and (C) represents the mean ± SD of at least ten replicates. n.s. $P > 0.05$. *$P < 0.05$. (Student's *t*-test of individual OE lines versus WT).

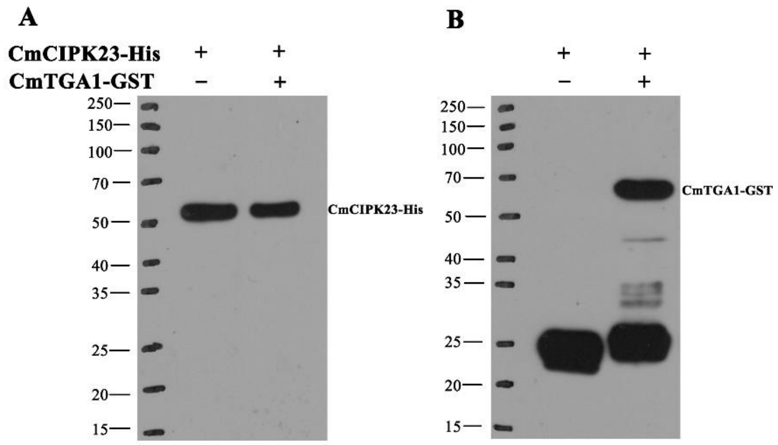

Fig. S4 Western blot detection of the interaction between CmCIPK23 and CmTGA1 in a His pull-down assay. (A) Anti-His antibody was used to detect CmCIPK23-His immobilization. (B) Anti-GST antibody was used to detect the CmTGA1-GST bound by the indicated CmCIPK23-His protein.

Table S1. The list of primers used in this study.

Primer name	Forward primer	Reverse primer
GSP3′	CCTCCCTGGTTCTCCGCAAGT	
GSP5′-1		TGTCACCTCCAGCCTTGCGAACCTCA
GSP5′-2		ATCCCAAAGGTCCAGCAGCTTCTTCA
CIPK23	ATGTCGAGTAGACCGTCTCG	TTACGAAGGTAATGGAGCACC
CIPK23 (qPCR)	AGCCGGGGTGTTTTCCATAG	TGTTGTGGGAGTGCACTCAA
CmUbi	CTAATGAATGCTTACTGTGACCGAC	AGGCGAATCATCAGTACCAAGTG
CIPK23-1258	AATGTCGAGTAGACCGTCTCGAACTC	CGAAGGTAATGGAGCACCTGC
CIPK23-PET28a	gaattcATGTCGAGTAGACCGTCTCGAACTC	aagcttCGAAGGTAATGGAGCACCTGC
TGA1-pGEX-6P-1	ggatccATGAATTCATCGTCGACTC	gaattcCAAAGCCGGTTCACAAA
CIPK23-BiFc	tctagaATGTCGAGTAGACCGTCTCG	cccgggCGAAGGTAATGGAGCACCTG
TGA1-BiFc	ggatccATGAATTCATCGTCGACTC	ctcgagCAAAGCCGGTTCACAAA
AtUBQ5	GGTGCTAAGAAGAGGAAGAAT	CTCCTTCTTTCTGGTAAACGT
AtHHO1	TGCGAGTACATCGAAGCTCTCG	CGACGTCCCTGATATCTCCCTC
AtHRS1	CAACCCGACCACCCACTT	TTCGCCACCGTTCACCAT
AtNIR	TATCGGATTCATGGGATGCT	AATGGAACCAACTCCGTGAC
AtNRT2.1	CGGAAAGATTCTGTGGTAT	GCTTCAAGTGAAACCTGTC
AtNIA1	CGAAGCTTGGTGGTATAA	ACCTCCTCCAGAATAAGC
AtNIA2	GAATGTTGGCAGGTGGAAC	GTATTGCTCTGCCCAACCAT
AtGS2	CGAGTACCGGTCAAGCACCT	TGCCTCCACGGAAAGGATCT
